# Cytotoxicity of Essential Oil *Cordia verbenaceae* against *Leishmania brasiliensis* and *Trypanosoma cruzi*

**DOI:** 10.3390/molecules26154485

**Published:** 2021-07-25

**Authors:** Pedro S. Pereira, Carlos Vinicius B. Oliveira, Ana J. Maia, Saulo R. Tintino, Cícera Datiane de M. Oliveira-Tintino, Maria C. Vega-Gomez, Miriam Rolón, Cathia Coronel, Antônia Eliene Duarte, Luiz M. Barros, Jeam Paul Kamdem, Abolghasem Siyadatpanah, Polrat Wilairatana, Henrique D. M. Coutinho

**Affiliations:** 1 Laboratório de Farmacologia e Química Molecular, Universidade Regional do Cariri, Rua Cel. Antonio Luis 1161, Pimenta, Crato 63105-000, Brazil; pedro.sillvino@gmail.com (P.S.P.); viniciusbluesky@gmail.com (C.V.B.O.); anajosicleide.maia@gmail.com (A.J.M.); duarte105@yahoo.com.br (A.E.D.); lmarivando@gmail.com (L.M.B.); kamdemjeanpaul2005@yahoo.fr (J.P.K.); 2 Centro para el Desarrollo de la Investigación Científica (CEDIC), Fundación Moisés Bertoni/Laboratorios Díaz Gill., Asunción 1255, Paraguay; saulorelison@gmail.com (S.R.T.); datianemorais@gmail.com (C.D.d.M.O.-T.); mcvegagomez@gmail.com (M.C.V.-G.); rolonmiriam@gmail.com (M.R.); cathiacoronel@gmail.com (C.C.); 3 Ferdows School of Paramedical and Health, Birjand University of Medical Sciences, Birjand 9717853577, Iran; asiyadatpanah@yahoo.com; 4 Department of Clinical Tropical Medicine, Faculty of Tropical Medicine, Mahidol University, Bangkok 10400, Thailand; 5 Laboratório de Microbiologia e Biologia Molecular, Universidade Regional do Cariri, Rua Cel. Antonio Luis 1161, Pimenta, Crato 63105-000, Brazil

**Keywords:** anti-kinetoplastidea, alfa-pinene, caryophyllene, tricyclo[2.2.1-(2,6)]heptane, Chagas disease, essential oil, natural product

## Abstract

The species *Cordia verbenacea* DC (Boraginaceae), known as the whaling herb and camaradinha, is a perennial shrub species native to the Atlantic Forest. Its leaves are used in folk medicine as an anti-inflammatory, analgesic, antiulcerogenic and curative agent, in the form of teas or infusions for internal or topical use. The present study aimed to verify the cytotoxicity of the essential oil and the leishmanicidal and trypanocidal potential of *C. verbenacea*. The essential oil was characterized by GC-MS. The in vitro biological activity was determined by anti-Leishmania and anti-Trypanosoma assays. The cytotoxixity was determined using mammalian fibroblasts. The *C. verbenacea* species presented α-pinene (45.71%), β-caryophyllene (18.77%), tricyclo[2,2,1-(2.6)]heptane (12.56%) as their main compounds. The essential oil exhibited strong cytotoxicity at concentrations below 250 μg/mL (LC_50_ 138.1 μg/mL) in mammalian fibroblasts. The potent anti-trypanosome and anti-promastigote activities occurred from the concentration of 62.5 μg/mL and was considered clinically relevant. The results also demonstrate that at low concentrations (<62.5 μg/mL), the essential oil of *C. verbenacea* managed to be lethal for these activities. This can be considered an indication of the power used in daily human consumption. Therefore, it can be concluded that the essential oil of *C. verbenacea* contains a compound with remarkable antiparasitic activities and requires further research.

## 1. Introduction

Parasitic diseases are among some of the most devastating and prevalent infections, causing millions of morbidities and deaths annually, where one of the main causes are kinetoplastids [1]. The kinetoplastid class is a diverse group of single-celled flagellate organisms, many of which are human parasites, that commonly present a reduction in or total elimination of biochemical pathways that can be augmented or provided by their hosts [2,3]. Diseases ignored by parasites such as *Leishmania brasiliensis* and *Trypanosoma cruzi* (kinetoplastids) affect thousands of people worldwide, and so there is an ongoing effort to identify new chemotherapies [4,5].

Viable antileishmanial drugs still depend on high doses of pentavalent antimonials, such as glucantime and meglumine antimoniate, that cause severe side effects and require long-term treatment [6]. Pentavalent antimonials are rapidly absorbed after administration and are converted into trivalent antimonite, the active form of the drug, which occurs either in the macrophages or in the parasite [7,8,9]. A specific parasite enzyme (thiol-dependent reductase I-TDR1) is capable of catalyzing the conversion of the pentavalent form of antimony to the trivalent one using glutathione as the reducing agent [10,11]. The active mechanism of antimonials is based on the interference of amastigote bioenergetic processes, involved in glycolysis and fatty acid oxidation, and reports have raised the possibility that antimony could trigger apoptosis [12]. Recently, a variety of enzymes have become targets for the development of new therapies against leishimaniasis, such as: ornithine decarboxylase (ODC), dihydrofolate reductase (DHFR), thymidylate synthase (TS) and trypanothione reductase [13,14,15].

There are currently only two anti-trypanosome drugs marketed: nifurtimox (Lampit^®^, Bayer, Leverkusen, Germany), 3-methyl-4-(5′-nitrofurfurilideneamino) tetrahydro-4H-1,4-thiazine-1,1-dioxide; and benznidazole (Rochagan^®^, Roche, Basel, Switzerland), *N*-benzyl-2-nitroimidazole acetamide [16,17]. However, both drugs can have numerous side effects in adults, in addition to inducing resistance in microorganisms [18]. The most common side effects in treatment with nifurtimox are nausea, vomiting, epigastric pain and dysphagia. Adverse events that occur most frequently in treatment with benzonidazole are itching, rash and sensitive neuropathy [19,20]. Among the compounds found in plants, alkaloids, flavonoids, piperine, terpenes and naphthoquinones represent important classes of natural products that have been tested [21].

In this context, essential oils from various plant species have become potential chemotherapeutic agents due to their antiparasitic properties [22,23,24]. Essential oils are insoluble in inorganic solvents (water), while also soluble in organic solvents. They are volatile liquids, with a characteristic odor, generally present in medicinal plants and composed of a mixture of substances of different classes [25,26,27]. Essential oils are of increasing importance in pharmaceutical practices due to their wide range of applications and biological activities [28,29]. Among the numerous pharmacological properties of essential oils are: antimicrobial [30], antiparasitic [31,32], antiulcerogenic [33], insecticide [34] and antifungal [35] properties. They are also widely used in perfumery, aromatherapy and added to spices or herbs [36,37]. The antileishimanial and antiprypanosomal activity of essential oils acts mainly by inducing apoptosis and damaging the only mitochondria of microorganisms, respectively [38,39].

From this perspective, a species whose essential oil has a huge medicinal potential is *C. verbenaceae* DC (Family: Boraginaceae. Synonyms: *Varronia curissavica* Jacq., *Cordia curassavica* (Jacq.) Roem. & Schult. and *Cordia monosperma* Jacq. which has been used in the treatment of bladder diseases), a perennial plant commonly found throughout the Brazilian coast [40] and commonly known as “erva baleeira”. It is a medicinal herbaceous dicocotyledonous plant not endemic in Brazil. Its globular trichomes are characterized by the secretion of a terpenoid essential oil, while in reniforms it contains mainly phenolic compounds, such as flavonoids [41,42].

The α-pinene, α-santalene and (E)-cariophyllene are the representative compounds present in the essential oil of *C. verbenaceae* [43,44]. The economic and pharmacological potential of this essential oil is due to the production of the chemical markers β-caryophyllene and α-humulene [40], which are associated with cytotoxicity and antiproliferative, pro-apoptotic and antitumor activities [45].

The study of *C. verbenaceae* in this research is due to its wide popular use and several biological activities mentioned above. The present study aimed to verify the leishmanicidal and trypanocidal power, as well as the cytotoxicity in vitro of the *C. verbenaceae* essential oil.

## 2. Results

According to Table 1, the chemical composition of the *C. verbenaceae* essential oil presented α-cymene, Caryophyllene and Tricyclo[2.2.1.0(2,6)]heptane as the major constituents, followed by other compounds at lower concentrations.

**Figure 1:**
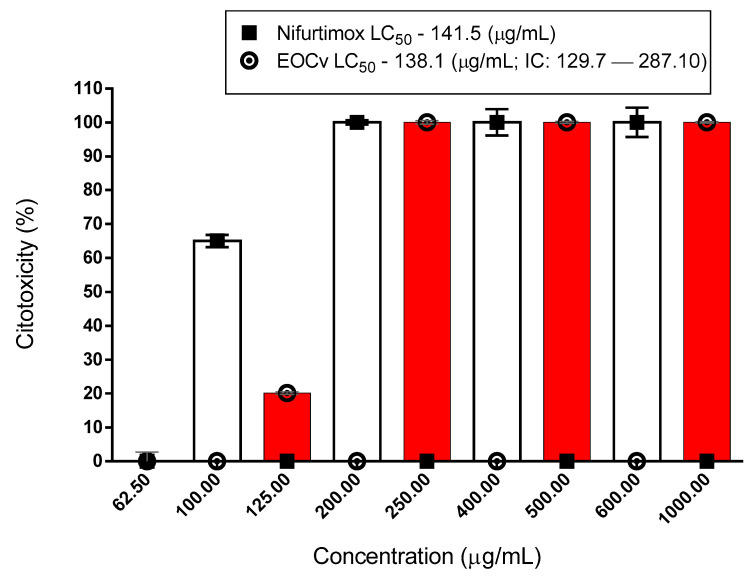
Cytotoxicity of *C. verbenaceae* essential oil. EOCv (essential oil of *C. verbenaceae*).

**Figure 2:**
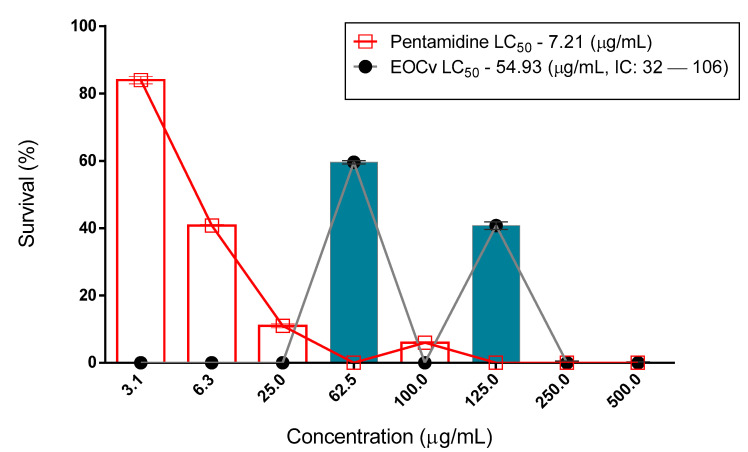
Survival of the promastigote *L. brasiliensis* treated with *C. verbenaceae* essential oil. LC_50_ confidence interval for oil 95% (32–106). EOCv (essential oil of *C. verbenaceae*).

**Figure 3:**
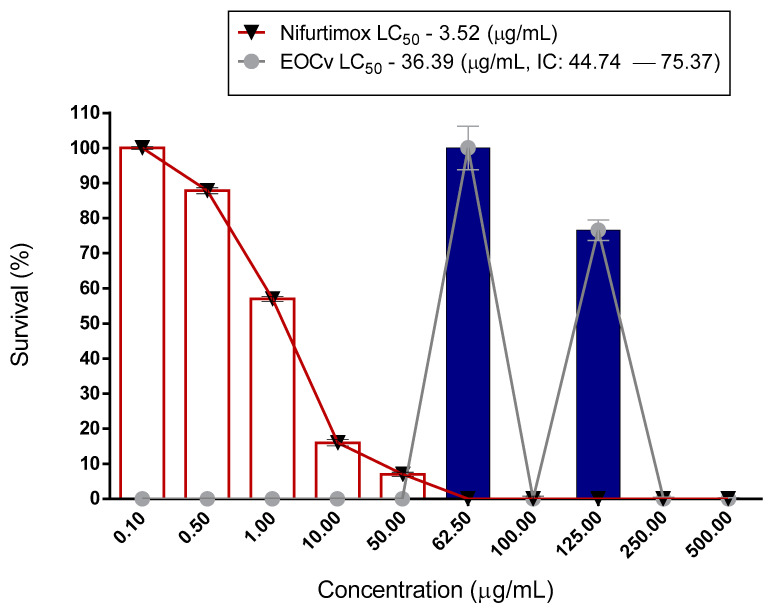
Survival of the epimastigote *T. cruzi* treated with *C. verbenaceae* essential oil. LC_50_ confidence interval for oil: 95% (44.74–75.37). EOCv (essential oil of *C. verbenaceae*).

The cytotoxic activity had an LC_50_ of 138.1 μg/mL (Figure 1 and Table 2). According to Figure 2 and Figure 3, and Table 3 and Table 4, the essential oil of *C. verbenaceae* exhibited values considered relevant for the promastigote and epimastigote forms of *L. brasiliense* and *T. cruzi*, with LC_50_ values of 67.18 μg/mL for *Leishmania* and 92.01 μg/mL for *Trypanosome*.

Table 2 shows that the essential oil of *C. verbenacea* is cytotoxic from a concentration of 125 µg/mL and at low concentrations (31.5 and 62.5 µg/mL) it has low cytotoxicity, which may be an indication of future human consumption. The oil was lethal at these concentrations to *L. brasiliensis* and *T. cruzi*.

According to Table 3, the EOCv at a concentration of 31.5 µg/mL, managed to be lethal at approximately 70%, thus presenting a good anti-leishmanial activity.

## 3. Discussion

Vegetables have been the object of study and biological screening as an alternative for the treatment of neglected parasitic diseases [46,47]. Phytochemicals are currently being synthesized and chemically modified to ensure greater potency against these human pathogens [48]. In this context, the main objective of this study was to investigate the biological activities of the essential oil of *C. verbenacea,* in order to investigate its potential to inhibit the promastigote forms of *Leishmania braziliensis* and the epimastigote forms of *Trypanosoma cruzi*.

Essential oils are complex mixtures consisting of terpenoid hydrocarbons, oxygenated terpenes and sesquiterpenes. They originate from the secondary metabolism of the plant and are responsible for its characteristic aroma. Gas chromatography (GC) is the best method, due to its simplicity, speed and efficiency, both for the identification and quantification of the components of essential oils and variations in composition [49].

Within the *Cordia genus*, there is a certain variation in the predominance of certain components of essential oils. Some species mainly contain monoterpene hydrocarbons common to *C. cylindrostachia* [50], *C. globosa* [51]; some sesquiterpene hydrocarbons, as seen in *C. verbenacea* [6], *C. leucocephala* [52] and *C. millenii* [53]; a mixture of monoterpene hydrocarbons and sesquiterpenes prominent in *C. trichotoma* [54] and *C. multispicata* [55]; oils containing oxygenated monoterpenes/sesquiterpenes, such as *C. myxa* [56] and *C. chacoensis* [57], respectively; and oils with a high content of non-terpenes, present in *C. sebestena* [58] and *C. gilletii* [59].

Regarding the characteristic chemical composition of the oil in the present study, it showed similarities between most studies observed so far. The α-pinene compound was detected in much larger quantities than the others, corroborating many other similar studies [60,61]. Caryophyllene and α-pinene demonstrated anti-trypanocidal action against *T. cruzi* in the epimastigote form [62]. Still, in relation to the essential oil from *C. verbenaea*, some studies have been conducted relating its action to anti-trypanocidal and anti-*Leishmania* [63]. The class of compounds present in the greatest quantity are hydrocarbon monoterpenes, followed hydrocarbon sesquiterpenes, which is consistent with previous findings [44]. Regarding the compound Tricyclo 2.2.1 (2,6) heptane, this is the first report of its presence in this species.

Previous evaluations have shown that the IC_50_ for NCTC929 fibroblasts exposed to essential oils is generally greater than 300 μg/mL, which indicates that the *C. verbenacea* essential oil demonstrates great viability as a leishimanicidal and trypanocidal agent in vivo [46,64].

According to Ibrahim et al. [65], the fruit pulp extract of *Cordia dichotoma* was able to inhibit the development of the breast cancer cell line (MCF7), an effect similar to that found in the work of Ashmawy et al. [66], where the essential oil of *C. africana* exhibited a potent cytotoxicity against the same cell line, probably through the regulation of apoptosis. Studies on the toxicity of the *C. verbenacea* essential oil are practically non-existent, but it is known that the leaf extract of these species can reduce the cell viability of MCF7, presenting an IC_50_ = 154 μg/mL [45].

The anti-leishmanicidal activity of the essential oil of *C. verbenacea* was evaluated against the promastigote form of *Leishmania braziliensis*. At a concentration of 125 μg/mL, the essential oil of *C. verbenacea* exhibited 59.22% anti-promastigote activity and was more effective against *L. braziliensis* at a concentration of 250 μg/mL, whose activity was 100%. The essential oil of *C. verbenacea* showed an IC_50_ = 54.93 μg/mL, demonstrating that it was less active than the positive pentamidine control (IC_50_ = 7.21 μg/mL) (Figure 2 and Table 3). The difference in the effectiveness of these oils against different species of Leishmania can be attributed to their distinct chemical compositions [46].

The values referring to Figure 2 and Table 3 are considered clinically relevant, as they had an effect at a concentration below 300 μg/mL, with an LC_50_ = 54.93 μg/mL, a more promising result than usual [32]. Studies on the activity of the antipromastigote potential of this species practically do not exist, although they can be found for other species of the same genus [67]. Regarding their constituents, there are many studies that show their actions. This is true in the case of α-pinene, which has already been shown to be an effective leishmanicidal agent due to the activity observed against the promastigote forms of *L. major*, as well as other hydrocarbon monoterpenes [68].

In relation to the other main constituent, caryophylene, according to the study by Moreira et al. [22], this compound showed activity against *Leishmania amazonensis*, in the promastigote and amastigote forms, with an IC_50_ of 49.9 μg/mL and 10.7 μg/mL, respectively. In the study by Ghaderi et al. [69], it is indicated that the caryophylline oxide compound acts to inhibit oxygen consumption by *Leishmania tarentolae* mitochondria, causing increased oxidative stress, decreased ATP production and the consequent death of the parasite.

The potential of the *C. verbenacea* essential oil to inhibit the epimastigote form of *Trypanosoma cruzi* was pioneered in this study (Figure 3). At the same time the *C. verbenacea* essential oil was highly toxic (100% lethal) to NCTC929 fibroblasts. The toxicity of the essential oil of *C. verbenacea* was possibly related to its monoterpenes (α-pinene) and hydrogenated sesquiterpenes (caryophyllene, tricyclo[2.2.1-(2,6)]heptane) present in the leaves of the plant species in this study. In this follow-up, we demonstrated that (*E*) -cariophylene exhibited a potent antiparasitic effect against *T. cruzi*, the result of which suggested that this compound may be the one that was responsible for the antiparasitic activity revealed in this investigation [70]. However, we cannot disregard a possible synergistic effect of the minor and major compounds of the essential oil of *C. verbenacea*.

The analysis of the result shown in Figure 3 and Table 4 revealed that the essential oil of *C. verbenacea* also presented values considered relevant, according to Meira et al. [71], which considered relevant IC_50_ values below 41.3 μg/mL, whereas the present study was 36.39 μg/mL. With regard to the epimastigote activity of the essential oil of this plant, this study is considered to have been pioneering. In addition, there are only studies on compounds identified after isolation. In the research carried out by Kamte et al. [72], the effect of α-Pinene was tested against the epimastigote form of *T. brucei* (TC221), where an LC_50_ of this compound equal to 1.0 μg/mL was observed. As for the other compound, the main caryophyllene constituent, it has already been shown to be active against the epimastigote, amastigote and promastigote forms of *T. cruzi* [73].

Essential oils and their constituents can act on parasites of the genus Trypanosoma and Leishmania in several ways: (1) They affect the layers of polysaccharides, fatty acids and phospholipids in the autophagosomal structures, causing disturbances in the nuclear membrane and in the condensation of the nuclear chromatin; (2) they interrupt specific metabolic pathways for lipids and proteins or stimulate the depolarization of mitochondrial membranes, which can lead to cell necrosis or apoptosis [74]; and (3) they increase reactive oxygen species that cause DNA damage [75].

## 4. Materials and Methods

### 4.1. Material Vegetal

The leaves of *C. verbenaceae* were collected at Crato Seedling Production Bank (voucher number # 044171).

### 4.2. Obtaining Essential Oil

The fresh leaves were cut into 1 cm^2^ pieces, washed and macerated with 99.9% ethanol for 72 h at room temperature. The essential oil was obtained by hydrodistillation in a Clevenger-type apparatus. Fresh leaves of *C. verbenaceae* were placed in a 5 L flask, together with 3 L of distilled water and heated for 2 h. Afterward, the water/oil mixture obtained was separated, and the essential oil of *C. verbenaceae* was treated with anhydrous sodium sulfate, filtered and kept under refrigeration until the time of analysis.

### 4.3. Gas Chromatography Mass Spectrometry Analysis (GC MS)

Oil analysis was performed using a Shimadzu GC MS-QP2010 series (GC/MS system): Rtx-5MS capillary column (30 m × 0.25 mm, 0.25 μm film thickness); helium carrier gas at 1.5 mL/min; injector temperature 250 °C; detector temperature 290 °C; column temperature 60–180 °C at 5 °C /min, then 180–280 °C at 10 °C/min (10 min). Scanning speed was 0.5 scan/sec from *m*/*z* 40 to 350. Split ratio (1:200). Injected volume: 1 µL of [25 µL (essential oil)/5 mL CHCl_3_] (1:200). Solvent cut time = 2.5 min. The mass spectrometer was operated using 70 eV of ionization energy. Identification of individual components was based on their mass spectral fragmentation based on Mass spectral library NIST 08, retention indices, and comparison with published data.

### 4.4. Antiparasitic Activity

#### Cell Lines Used

Strains of CL-B5 parasites (clone CL-B5) were used for in vitro evaluation of the activity on *T. cruzi* [76]. Parasites transfected with the β-galactosidase gene of *Escherichia coli* (LacZ) were provided by Dr. F. Buckner through the Gorgas Memorial Institute (Panama). Epimastigotes forms cultured in LIT infusion tryptose liver at 28 °C plus 10% fetal bovine serum (FBS), penicillin 10 U/mL and 10 μg/mL streptomycin at pH 7.2, were incubated with different concentrations of essential oil (125, 62.5, 31.25 and 15.62 μg/mL) and harvested during the exponential growth phase [77].

Antileishmanial in vitro activity was determined using promastigotes of *L. braziliensis* (MHOM/CW/88/UA301) at 26 °C, grown in Schneider’s insect medium, supplemented with 10% (*v*/*v*) heat-inactivated fetal calf serum, 2% normal human urine (*v*/*v*) plus penicillin and streptomycin [33]. The forms were seeded and incubated with different concentrations of essential oil (125, 62.5, 31.25 and 15.62 mL).

### 4.5. Reagents

The sodium resazurin substance was obtained from Sigma-Aldrich (St. Louis, MO, USA) and stored at 4 °C protected from light. A resazurin solution was prepared with 1% phosphate buffer, at pH 7, and was sterilized in advance by filtration. Afterward, the chlorophenol red-β-d-galactopyranoside-CPRG (Roche, Indianapolis, IN, USA) was dissolved in a solution of Triton X-100 0.9% (pH 7.4). Penicillin G (Ern, SA, Barcelona, Spain), streptomycin (Reig Jofre SA, Barcelona, Spain) and Dimethyl sulfoxide (DMSO) were also used.

### 4.6. In Vitro Epimastigote Susceptibility Assay

The assays were performed according to the procedures described by Vega et al. [78], with crops that had not reached the stationary phase. Epimastigotes forms were seeded at 1 × 10^5^ per mL in 200 μL, in 96-well microdilution plates, which were incubated at 28 °C for 72 h. Then, 50 μL of CPRG (Chlorophenol red-β-D-galactopyranoside) solution was added to give a final concentration of 200 μM. The plates were incubated at 37 °C for an additional 6 h. The absorbance reading was performed in a spectrophotometer at 595 nm. Nifurtimox was used as reference drug. The concentrations were tested in triplicate. Each experiment was performed twice separately. The inhibition percentage (% AE) was calculated as follows:

% AE = [(AE_AEB)/(AC_ACB)] × 100(1)

where AE represents the “absorbance of tested plates,” AEB is the “absorbance of plates containing medium and sample,” AC is the “absorbance of plates containing negative control,” and ACB is the “absorbance of plates containing culture medium.”

All the IC_50_ values were calculated by a nonlinear regression equation, using the computer program GraphPad Prism v. 6.0. The concentration of DMSO (dimethyl sulfoxide) used to enable oil solubility was not greater than 0.01%.

### 4.7. In Vitro Leishmanicidal Assay

The assays were performed according to the procedures described by Mikus and Steverding [79] with some adjustments. The activity of the oil was performed in triplicate. Promastigotes forms (2.5 × 10^5^ parasites/well) were cultured in 96-well plastic plates. The samples were dissolved in dimethylsulfoxide (DMSO). Different dilutions of the compounds, up to 200 mL of the final volume, were added. After 48 h at 26 °C, 20 µL of resazurin solution was added and the oxidation–reduction was measured from 570 to 595 nm. In each assay pentamidine was used as a control reference drug. The anti-promastigotes percentages (AP%) were calculated. The efficacy of each compound was determined.

### 4.8. Cytotoxic Assays

In order to measure the cell viability, a colorimetric assay with resazurin was used [80]. NCTC 929 fibroblasts were seeded (5 × 10^4^ cells/well) in flat-bottom microdilution plates of 96 wells with 100 μL of RPMI 1640 medium for 24 h at 37 °C in 5% CO_2_ for the cells to adhere to the plates. The medium was replaced by different concentrations of drugs in 200 μL of medium and incubated for another 24 h. Growth controls were included. Then, a volume of 20 μL of 2 mM solution of resazurin was added and the plates were placed in the incubator for another 3 h to assess cell viability. The reduction of resazurin was determined by measuring wavelength absorbance between 490 nm and 595 nm during the test’s controls with media where drugs were used. Each concentration was tested three times. The cytotoxicity of each compound was estimated by calculating the percentage of cytotoxicity (% C).

### 4.9. Statistical Analysis

Results were expressed as mean ± standard error of the mean (SEM) of three independent experiments carried out in triplicate. The LC_50_ values were calculated, by Linear Regression, using the GraphPad Prism software version 6.0.

## 5. Conclusions

As seen during this research, the essential oil of *C. verbenaceae* proved to be effective in both *Leishmania* and *Trypanocida* activities. In the trypanocidal activity, the oil of *C. verbenacea* in low concentration was lethal, with low cytotoxicity. The values of this oil presented here can be considered of clinical relevance, because parasitic lethality occurs at low concentrations. This result may support its future clinical application, since one of the main problems in the use of essential oils is toxicity.

## Figures and Tables

**Table 1:** Chemical composition (%) of the essential oil of *C. verbenacea*.

Components	RT (min) ^a^	(%)
α-thujene α-pinene Zingiberene Tricyclo[2.2.1.0(2,6)]heptane Caryophyllene	12.09 12.52 39.91 40.90 41.13	1.70 45.71 1.37 12.56 18.77
α-humulene	43.24	3.43
Alloaromadendrene	43.77	4.37
Beta-bisabolene	46.10	3.53
Tetradecane Nerolidol Caryophyllene oxide α-sinensal Santalol	47.86 52.70 47.98 49.04 49.15	1.19 3.38 2.04 2.43 2.90
Total		100.00

Averages followed by different letters differ by Tukey test at *p* < 0.05. ^a^ Retention time.

**Table 2:** Cytotoxicity of *C. verbenacea* essential oil.

Natural Product	Conc. µg/mL *C. verbenacea*	%C	±%DS	Conc. µg/mL Nifurtimox	%C	±%DS
*C. verbenacea*	1000	100	-			
				600	100	4.3
	500	100	-			
				400	100	3.9
	250	97.85	0.49			
				200	100	0.6
	125	64.21	0.80			
				100	65	1.8
	62.5	10.04	0.70			
	31.5	9.04	0.77			

**Table 3:** Survival of the promastigote *L. brasiliensis* treated with *C. verbenaceae* essential oil.

Natural Product	Conc. µg/mL *C. verbenacea*	%S	±%DS	Conc. µg/mL Pentamidine	%S	±%DS
*C. verbenacea*	1000	100	-			
	500	100	-			
	250	100	-			
	125	100	-			
				100	6.0	0.3
	62.5	60.87	2.11			
	31.5	34.44	1.02			
				25	11	0.6
				6.2	40.8	0.3
				3.2	84	1.1

**Table 4:** Survival of the epimastigote *T. cruzi* treated with *C. verbenaceae* essential oil.

Natural Product	Conc. µg/mL *C. verbenacea*	%S	±%DS	Conc. g/mL Pentamidine	%S	±%DS
*C. verbenacea*	1000	100	-			
	500	100	-			
	250	100	-			
	125	100	-			
				100	0	0.8
	62.5	25.11	1.70			
				50	7	0.6
	31.5	16.12	2.52			
				10	16	0.9
				1.0	57	0.7
				0.5	87	0.8
				0.1	100	0.4

## Data Availability

Data is contained within the article.

## Sample Availability

Samples of the compounds are available from the authors.
